# Peri-Operative Prophylaxis in Patients of Neonatal and Pediatric Age Subjected to Cardiac and Thoracic Surgery: A RAND/UCLA Appropriateness Method Consensus Study

**DOI:** 10.3390/antibiotics11050554

**Published:** 2022-04-21

**Authors:** Sonia Bianchini, Laura Nicoletti, Sara Monaco, Erika Rigotti, Agnese Corbelli, Annamaria Colombari, Cinzia Auriti, Caterina Caminiti, Giorgio Conti, Maia De Luca, Daniele Donà, Luisa Galli, Silvia Garazzino, Alessandro Inserra, Stefania La Grutta, Laura Lancella, Mario Lima, Andrea Lo Vecchio, Gloria Pelizzo, Nicola Petrosillo, Giorgio Piacentini, Carlo Pietrasanta, Nicola Principi, Matteo Puntoni, Alessandro Simonini, Simonetta Tesoro, Elisabetta Venturini, Annamaria Staiano, Fabio Caramelli, Gaetano Domenico Gargiulo, Susanna Esposito, on behalf of the Peri-Operative Prophylaxis in Neonatal and Paediatric Age (POP-NeoPed) Study Group

**Affiliations:** 1 Pediatric Clinic, University Hospital, Department of Medicine and Surgery, University of Parma, 43126 Parma, Italy; bianchini.sonia@outlook.it (S.B.); laura.nicoletti@studenti.unipr.it (L.N.); s.monaco1410@gmail.com (S.M.); 2 Pediatric Clinic, Azienda Ospedaliera Universitaria Integrata, 37134 Verona, Italy; erika.rigotti@aovr.veneto.it (E.R.); agnesecorbelli@gmail.com (A.C.); anna_maria.colombari@studenti.univr.it (A.C.); giorgio.piacentini@univr.it (G.P.); 3 Neonatology and Neonatal Intensive Care Unit, IRCCS Bambino Gesù Children’s Hospital, 00165 Rome, Italy; cinzia.auriti@opbg.net; 4 Clinical and Epidemiological Research Unit, University Hospital of Parma, 43126 Parma, Italy; ccaminiti@ao.pr.it (C.C.); mpuntoni@ao.pr.it (M.P.); 5 Pediatric ICU and Trauma Center, Fondazione Policlinico Universitario A. Gemelli IRCCS, 00165 Rome, Italy; giorgio.conti@unicatt.it; 6 Paediatric and Infectious Disease Unit, Academic Department of Pediatrics, IRCCS Bambino Gesù Children’s Hospital, 00165 Rome, Italy; maia.deluca@opbg.net (M.D.L.); laura.lancella@opbg.net (L.L.); 7 Division of Paediatric Infectious Diseases, Department for Woman and Child Health, University of Padua, 35100 Padua, Italy; daniele.dona@unipd.it; 8 Pediatric Infectious Diseases Unit, Meyer’s Children Hospital, 50139 Florence, Italy; luisa.galli@unifi.it (L.G.); elisabetta.venturini@meyer.it (E.V.); 9 Pediatric Infectious Diseases Unit, Regina Margherita Children’s Hospital, University of Turin, 10122 Turin, Italy; silvia.garazzino@unito.it; 10 General Surgery Department, IRCCS Bambino Gesù Children’s Hospital, 00165 Rome, Italy; alessandro.inserra@opbg.net; 11 Institute of Translational Pharmacology IFT, National Research Council, 90146 Palermo, Italy; stefania.lagrutta@cnr.it; 12 Pediatric Surgery, IRCCS Azienda Ospedaliera-Universitaria di Bologna, 40138 Bologna, Italy; mario.lima@unibo.it; 13 Department of Translational Medical Science, Section of Pediatrics, University of Naples “Federico II”, 80138 Naples, Italy; andrealovecchio@gmail.com; 14 Pediatric Surgery Department, “Vittore Buzzi” Children’s Hospital, 20154 Milan, Italy; gloria.pelizzo@unimi.it (G.P.); staiano@unina.it (A.S.); 15 Infectious Disease and Infection Control Unit, Campus Bio-Medico, Medicine University Hospital, 00128 Rome, Italy; n.petrosillo@unicampus.it; 16 NICU, Fondazione IRCCS Ca’ Granda Ospedale Maggiore Policlinico, Department of Mother, Child and Infant, 20122 Milan, Italy; carlo.pietrasanta@unimi.it; 17 Università degli Studi di Milano, 20122 Milan, Italy; nicola.principi@unimi.it; 18 Pediatric Anesthesia and Intensive Care Unit, Salesi Children’s Hospital, 60123 Ancona, Italy; dr.simonini@gmail.com; 19 Division of Anesthesia, Analgesia, and Intensive Care, Department of Surgical and Biomedical Sciences, University of Perugia, 06129 Perugia, Italy; simonettatesoro@gmail.com; 20 General and Pediatric Anesthesia and Intensive Care Department, IRCCS Azienda Ospedaliero-Universitaria di Bologna, 40138 Bologna, Italy; fabio.caramelli@aosp.bo.it; 21 Department of Cardio-Thoracic and Vascular Medicine, Adult Cardiac Surgery, IRCCS Azienda Ospedaliero-Universitaria di Bologna, 40138 Bologna, Italy; gaetano.gargiulo@unibo.it

**Keywords:** antibiotics, cardiac surgery, ECMO, thoracic surgery, pediatric infectious diseases, surgical antibiotic prophylaxis

## Abstract

Surgical site infections (SSIs) represent a potential complication of surgical procedures, with a significant impact on mortality, morbidity, and healthcare costs. Patients undergoing cardiac surgery and thoracic surgery are often considered patients at high risk of developing SSIs. This consensus document aims to provide information on the management of peri-operative antibiotic prophylaxis for the pediatric and neonatal population undergoing cardiac and non-cardiac thoracic surgery. The following scenarios were considered: (1) cardiac surgery for the correction of congenital heart disease and/or valve surgery; (2) cardiac catheterization without the placement of prosthetic material; (3) cardiac catheterization with the placement of prosthetic material; (4) implantable cardiac defibrillator or epicardial pacemaker placement; (5) patients undergoing ExtraCorporal Membrane Oxygenation; (6) cardiac tumors and heart transplantation; (7) non-cardiac thoracic surgery with thoracotomy; (8) non-cardiac thoracic surgery using video-assisted thoracoscopy; (9) elective chest drain placement in the pediatric patient; (10) elective chest drain placement in the newborn; (11) thoracic drain placement in the trauma setting. This consensus provides clear and shared indications, representing the most complete and up-to-date collection of practice recommendations in pediatric cardiac and thoracic surgery, in order to guide physicians in the management of the patient, standardizing approaches and avoiding the abuse and misuse of antibiotics.

## 1. Introduction

Surgical site infections (SSIs) represent a potential complication of surgical procedures, with a significant impact on mortality, morbidity, and healthcare costs [1,2,3,4]. The type of surgery, its duration, the preoperative preparation, and the patient’s underlying conditions influence the incidence and severity of SSIs [5]. The use of preventive measures, foremost among which is the peri-operative administration of antibiotics, can effectively reduce the occurrence of SSIs, both in adults and pediatrics [6,7]. Most of the knowledge about the risk factors and prevention of SSIs relates to studies on adults, whereas currently available data on the pediatric and neonatal population are limited, resulting in difficulties in the management of SSI prophylaxis. 

In the field of cardiothoracic surgery, the rate of SSIs is a relevant issue. In the USA, the incidence of SSIs after cardiothoracic surgery ranges between 0.25 and 6%, with an associated mortality of 7–20%. In addition, the rate of postoperative nosocomial infection reaches values close to 23% [4,8,9]. The microorganisms isolated in SSIs of patients undergoing cardiothoracic procedures are usually skin pathogens such as *Staphylococcus aureus* and *Staphylococcus epidermidis*, while those responsible for postoperative pneumonia, an infectious complication of thoracic surgery, are mainly Gram-positive bacteria (*Streptococcus* spp. and *Staphylococcus* spp.); Gram-negative bacteria (*Haemophilus influenzae*, *Enterobacter cloacae*, *Klebsiella pneumoniae*, Acinetobacter spp., *Pseudomonas aeruginosa,* and *Moraxella catarrhalis*); and fungal pathogens (*Candida* spp.) [5,10,11,12]. 

Peri-operative risk factors for SSIs in the pediatric and neonatal population undergoing cardiothoracic surgery are: age less than 1 month, low birth weight, mechanical ventilation >3 days, duration of surgery, presence of genetic abnormalities, prolonged use of extracorporeal circulation (*ExtraCorporeal Membrane Oxygenation*, ECMO), delayed sternal closure, prolonged hospital stay and post-operative stay, post-operative hemorrhage, and persistent low cardiac output [13,14,15,16,17]. The risk of SSI is also increased by the frequent need to perform prolonged invasive procedures that cause increased bleeding and by the use of external devices (e.g., chest catheters, central venous or arterial catheters, and pacing wires), which may then remain in place postoperatively [18,19]. In particular, sternal wound infections (including mediastinitis), endocarditis, and infections of implanted prostheses (e.g., valves or other prosthetic material) can occur, with major sequelae, resulting in poor clinical outcomes, increased healthcare costs, and mortality [20].

Pediatric patients are particularly susceptible to SSIs due to a number of age-specific factors, including: immature renal function, altered circulatory pathways, the immaturity of the immune system, the use of deeper hypothermia to induce circulatory arrest, the complexity of reconstructive surgeries, the prolonged permanence of drainage tubes, and difficulty with post-operative renourishment [10,21,22]. This consensus document aims to provide information on the management of peri-operative antibiotic prophylaxis for the pediatric and neonatal population undergoing cardiac and non-cardiac thoracic surgery. 

## 2. Methods

### 2.1. RAND/UCLA Method of Appropriateness

This document was created using the RAND/UCLA (Research and Development Corporation of the University of California—Los Angeles) method of appropriateness. This method consists of a panel of experts evaluating the appropriateness of diagnostic, management, and therapeutic procedures with suboptimal scientific evidence [23]. According to the RAND method, a procedure is defined as “appropriate” if the expected benefits outweigh the expected negative consequences. Conversely, a procedure whose expected risks exceed its expected benefits is defined as inappropriate. According to the RAND definition, experts must make a judgment of appropriateness/inappropriateness by considering only the clinical benefits, without making economic considerations [24]. For a heterogeneous topic such as surgical antimicrobial prophylaxis, on which randomized controlled trials in pediatrics are lacking, the application of methods aiming to increase the homogeneity of behaviors by neonatologists, infectious disease specialists, pediatric surgeons, and anesthetists appeared useful and appropriate. For this reason, the RAND/UCLA approach was chosen instead of the GRADE methodology. Through the RAND method, the participants discussed different clinical scenarios and elaborated statements on the basis of the literature and their clinical experience. The group of experts did not consider it appropriate to combine the GRADE method with the RAND/UCLA approach, because the absence of randomized studies represents a bias in defining the strength of the recommendations and in representing a consensus reached for real life.

### 2.2. Recruiting the Expert Panel

A multidisciplinary group of experts belonging to the main Italian scientific societies composed of pediatricians, neonatologists, specialists in infectious diseases, pediatric surgeons, anesthesiologists, pharmacologists, and microbiologists was selected. The following Scientific Societies were involved: Italian Society of Pediatrics (SIP), Italian Society of Neonatology (SIN), Italian Society of Pediatric Infectious Diseases (SITIP), Italian Society of Infectious and Tropical Diseases (SIMIT), Italian Society of Pediatric Surgery (SICP), Italian Society of Microbiology (SIM), Italian Society of Pharmacology (SIF), Italian Society of Neonatal and Pediatric Anesthesia and Resuscitation (SARNEPI), and Italian Society of Childhood Respiratory Diseases (SIMRI). The panel of experts was made up of 52 medical doctors with at least 5 years of experience: pediatricians (n = 20), neonatologists (n = 6), infectious disease specialists (n = 5), pediatric surgeons (n = 5), anesthetists (n = 8), pharmacologists (n = 5), and microbiologists (n = 3).

### 2.3. Scenario Formulation

A literature search was performed with a selection of papers including randomized trials, systematic literature reviews, meta-analyses, and guidelines on peri-operative prophylaxis for the prevention of SSIs during cardiac surgery and thoracic surgery. The literature search was performed on the PubMed database, with a selection of English-language articles published from 2000 to 2020. In specific cases where recent literature was lacking, articles published since 1995 were also considered. Key search terms such as: “antimicrobial prophylaxis” OR “antibiotic prophylaxis” AND “thoracic surgery” OR “cardiac surgery” OR “heart pediatric surgery” OR “catheterization” OR “pacemaker implant” OR “sternotomy” OR “ECMO” OR “extracorporeal circulation” OR “congenital cardiopathy” OR “cardiac prosthesis” were used. Subsequently, using the Patient/Problem/Population-Intervention-Comparison/Control/Comparator-Outcome (PICO) model, a questionnaire on perioperative prophylaxis in cardiac and thoracic surgery was created for the pediatric and neonatal population and then divided into 11 clinical scenarios. Scenarios were identified on the basis of the clinical practice of the expert panel and answers included whether or not surgical antimicrobial prophylaxis (SAP) was recommended and, in the case of SAP recommendation, the antibiotic was considered as first choice. Before administration, the questionnaire was tested twice with a one-week interval to a convenience sample of 4 pediatricians, 2 neonatologists, 1 infectious diseases specialist, 1 pediatric surgeon, 1 anesthetist, 1 pharmacologist, and 1 microbiologist. Then, 26 out of 52 experts were selected by the Scientific Societies for answering, and the questionnaire was administered to 11 pediatricians, 3 neonatologists, 2 infectious diseases specialists, 3 pediatric surgeons, 4 anesthetists, 2 pharmacologists, and 1 microbiologist. 

### 2.4. Two-Round Consensus Process

Based on the scenarios, the questionnaire was submitted to experts on the online platform REDCap. REDCap (Research Electronic Data Capture) is a secure, web-based software platform designed to support data capture for research studies, providing: (1) an intuitive interface for validated data capture; (2) audit trails for tracking data manipulation and export procedures; (3) automated export procedures for seamless data downloads to common statistical packages; and (4) procedures for data integration and interoperability with external sources. Each question included the clinical scenario, and possible answers were whether or not SAP was recommended for the scenario and, in the case of its recommendation, a list with all the antibiotics available on the European market so that the expert could select the antibiotics that were considered by that individual as first choice. The selected bibliographic material was made available to all panel members, who were instructed on how to fill out the questionnaire. Experts responded to the questionnaire anonymously and their judgment was expressed on a scale from 1 to 9, where “1” was considered definitely inappropriate, “5” uncertain, and “9” definitely appropriate. The intermediate values corresponded to different modulations of the judgment of inappropriateness (“2” and “3”), uncertainty (from “4” to “6”), and appropriateness (“7” and “8”). In evaluating each indication, experts relied both on their own clinical judgment and experience and on available scientific evidence. Free space was provided for any annotations or comments. 

The first round of the questionnaire was conducted anonymously with respect to the other panel members. Multiple participation was not permitted by the platform, which also guaranteed the confidentiality and anonymity of the answers. The results of the survey were discussed in a collegial meeting with all the 26 experts who answered the questionnaire to reach agreements and reduce eventual disagreements [24]. Clarifications, adaptations, refinements of the guidance, and adequacy ratings were made. A total of 12 recommendations were developed. Participants were asked to endorse the recommendations in a second round during the following 4 weeks. 

## 3. Results

### 3.1. Cardiac Surgery

#### 3.1.1. SCENARIO #1: Cardiac Surgery for Correction of Congenital Heart Disease and/or Valve Surgery

Among pediatric operations, especially those within the first two years of life, the majority are performed for the correction of congenital cardiac anomalies and have excellent short- and long-term results [25]. Congenital heart disease is the leading cause of congenital anomalies and a significant global health problem, as 28% of all major anomalies are cardiac defects, with an incidence of approximately 8 per 1000 live births [26]. Table 1 shows the main congenital heart diseases.

The SSI rate in pediatric cardiac surgery should tend toward zero, as this is considered a “clean” type of surgery. However, at present, it ranges from 1% to 9%, with 23% as the highest rate of infection described in the literature [10,27,28,29].

##### Type of Molecule

The microorganisms involved in the SSIs of children undergoing this type of surgery are *Staphylococci* (especially *S. aureus* and coagulase-negative *Staphylococci*). Therefore, the molecules used for prophylaxis may be cephalosporins of I and II generation [14,21,30,31,32]. According to some authors, there seems to be no evidence of increased rates of Gram-negative infection, even in subjects with delayed sternal closure [10]. However, according to others, among pediatric subjects undergoing sternotomy, the presence of Gram-negatives, including *K. pneumoniae* and *P. aeruginosa*, is also frequently observed [31,32]. 

Antimicrobial perioperative prophylaxis is routinely given in this type of surgery, but the literature shows extreme heterogeneity, both in terms of the indication for prescription and in terms of the type and timing of molecules [33,34]. Jaworski’s group recently revealed a lack of rigorous clinical trials evaluating the safety and efficacy of antibiotic prophylaxis schedules in children undergoing cardiac surgery [30]. Al-Momany’s group highlighted that in some settings there is not optimal adherence to guidelines for peri-operative antimicrobial prophylaxis in cardiac surgery, resulting in the inappropriate use of different antibiotics [35]. Bratzler and colleagues specified that if the patient is already on antimicrobial therapy that is appropriate as a spectrum of action for prophylaxis, an extra dose should be administered 60 min before the incision; otherwise, prophylaxis considered appropriate for the type of surgery should be added [31]. The Society of Thoracic Surgeons guidelines recommended in the perioperative prophylaxis of adult patients undergoing cardiac surgery the use of I generation cephalosporin, i.e., cefazolin [12]. 

In patients where the sternum is left open to achieve better hemodynamic, respiratory, and hemostatic management of the pediatric patient, the risk of infection increases, resulting in high morbidity and mortality [36,37,38]. Hatachi’s work showed, in a sample of 63 pediatric patients undergoing cardiac surgery via sternotomy, a significant reduction in the incidence of SSIs and systemic infections with the combined use of vancomycin and meropenem compared with cefazolin regimens (alone or combined with vancomycin) [39]. These data support the absence of an advantage of vancomycin over cefazolin and the need to add vancomycin exclusively in settings with high MRSA prevalence [40,41]. 

**Recommendation 1.** In the pediatric and neonatal patient undergoing cardiac surgery for the correction of congenital heart disease and/or heart valve surgery via sternotomy or thoracotomy, peri-operative prophylaxis with cefazolin with a single dose of 30 mg/Kg (maximum dose 2 g) IV to be administered within 30 min before surgery is recommended. 

##### Intra-Operative Re-Dosing

It is well known that successful antimicrobial prophylaxis depends on both the selection of the appropriate molecule and its timing of administration to provide an adequate blood concentration before incision and maintain adequate levels throughout the procedure and immediately postoperatively. Time-dependent bacterial agents, such as cefazolin, must maintain a concentration above the minimum inhibitory concentration (MIC) for at least 40–50% of the dose interval [12,42]. The problem of cefazolin’s time dependence can be overcome by administering a higher initial dose, given the faster bactericidal capacity when levels are higher (due to the simultaneous binding to multiple penicillin-binding proteins) [43]. 

De Cock’s group evaluated the pharmacokinetics of cefazolin administered pre- and intra-operatively, reporting the following pattern: cefazolin 40 mg/Kg within 30 min of incision, 20 mg/Kg at the start of extracorporeal circulation (ECC), 20 mg/Kg at the start of rewarming from ECC, and 40 mg/Kg 8 h after the III and IV doses [44]. 

A 25 mg/Kg dose of cefazolin before incision and another 25 mg/Kg dose before ECC would appear to fail to achieve an effective serum concentration in younger children [45]. Most authors recommend an additional intraoperative dose every 3–4 h [30]. 

**Recommendation 2**. In the pediatric and neonatal patient undergoing cardiac surgery for the correction of congenital heart disease and/or heart valve surgery via sternotomy or thoracotomy, the addition of a second dose of cefazolin at a dose of 30 mg/kg (maximum dose 2 g) IV is recommended if the surgery lasts longer than 4 h. 

##### Continuing Post-Operative Prophylaxis

Knoderer’s group demonstrated in 210 pediatric subjects undergoing cardiac surgery that limiting the use of cefazolin to only 24 h after surgery, compared with its prolonged use, did not result in an increased incidence of infection [46]. Some authors consider it useful to continue peri-operative prophylaxis as long as chest drainage tubes are present or even until central venous catheters are removed [47], although most scientific evidence argues against there being an advantage to this practice [10,31]. In addition, many authors have shown that the discontinuation of antimicrobial prophylaxis within the first 48 h after surgery does not increase the incidence rate of infection [48]. In support of this, Alvarez’s group also reported an increased incidence of infection following the prolongation of prophylaxis beyond 48 h [27]. The Society of Thoracic Surgeons guidelines recommend prophylactic antibiotic use no longer than 48 h post-operatively [9]. Brocard’s group reported in a literature review that there is no evidence to support the prolongation of peri-operative prophylaxis in the post-operative setting, as was also reported in the 2018 guidelines by the World Health Organization (WHO) and the Pan American Health Organization (PAHO) [49,50,51]. 

**Recommendation 3**. In pediatric and neonatal patients undergoing cardiac surgery for the correction of congenital heart disease and/or heart valve surgery via sternotomy or thoracotomy, it is recommended to maintain prophylaxis with cefazolin at a dose of 30 mg/kg (maximum dose 2 g) every 8 until 24 h after sternum closure. This recommendation is independent of whether chest drains and vascular catheters remain in place. 

#### 3.1.2. SCENARIO #2: Cardiac Catheterization without Placement of Prosthetic Material 

Procedures involving cardiac catheterization can be divided into three main categories: diagnostic (assessment of anatomical structures and hemodynamics); therapeutic (e.g., ablation); and interventional (valvuloplasty, angioplasty, embolization, etc.). A further subdivision concerns whether or not prosthetic material is placed [52,53,54,55]. Infection rates with cardiac catheterization that does not involve the placement of prosthetic materiali are considered low, even with nondiagnostic catheterization [54,55,56,57]. 

The need for antibiotic prophylaxis in cardiac catheterization in the pediatric population remains undefined because of the lack of guidelines or systematic studies. Therefore, adult guidelines are usually applied, although this may be inappropriate because of the complexity and heterogeneity of the population. International guidelines covering the adult population consider cardiac catheterization procedures without device implantation as “clean” procedures and do not routinely recommend the administration of antibiotic prophylaxis [58]. However, peri-procedural antibiotic administration should be considered in immunocompromised patients and in those with probable or definite wound contamination during the procedure [58,59,60]. In cases where prophylaxis is required, the use of molecules effective against common skin pathogens (*S. aureus* and *S. epidermidis*) is recommended [5,27,28]. A 2015 retrospective study found that most specialists do not administer antibiotic prophylaxis in the case of procedures without the placement of prosthetic material. Specifically, the authors evaluated 215 pediatric cardiac catheterization procedures without the placement of prosthetic material, showing the absence of infectious complications both in patients who had not received antibiotic prophylaxis (approximately 64% of the total) and in those who had received a single dose of pre-procedural antibiotic. However, the authors, considering the small sample size analyzed, emphasized the need for further studies with larger cohorts [54]. 

**Recommendation 4**. In the pediatric patient undergoing diagnostic or interventional cardiac catheterization without prosthetic material placement, peri-operative antibiotic prophylaxis is not recommended. 

#### 3.1.3. SCENARIO #3: Cardiac Catheterization with Placement of Prosthetic Material

In recent decades, advances in the treatment of congenital heart disease have substantially reduced mortality and morbidity in both pediatric and adult populations [61,62,63]. Interventional catheterization with the use of endovascular stents, occluder devices, coils, or vascular plugs has contributed greatly to the successful management of children and adults with heart disease [64,65]. However, the implantation of prosthetic material exposes the patient to an increased risk of the occurrence of infectious complications, usually secondary to the implanted device rather than for complications at the surgical site [66,67,68,69,70,71,72]. 

The microorganisms most frequently involved are common skin pathogens, such as *S. aureus* or coagulase-negative *Staphylococci* [5,28]. Weber’s group examined 1085 pediatric patients undergoing cardiac catheterization and showed that the risk of infectious complications, particularly infective endocarditis, was related not to the procedure performed but to the placement of prosthetic material [70]. In the meta-analysis by Kreter and colleagues, the incidence of SSIs was evaluated by comparing the prophylactic administration of cephalosporins or penicillins, showing the greater efficacy of cephalosporins [73]. Both first- and second-generation cephalosporins were found to be effective, and randomized trials conducted to determine the molecule of choice were unable to define the superiority of one over the other [73,74,75]. A 2003 review recommends antibiotic prophylaxis in patients undergoing the placement of electrophysiologic cardiac devices (pacemakers, cardioverter defibrillators), ventricular assist devices, total artificial hearts, PTFE shunts, cardiac pledgets, vascular grafts, and vascular patches, with a single dose of peri-procedural antibiotic. One dose of cefazolin is usually used to prevent methicillin-susceptible staphylococcal infection; vancomycin should be considered for use only in patients who do not tolerate beta-lactam antibiotics or who are colonized or infected with methicillin-resistant *S. aureus* (MRSA). Additional doses of antibiotic may be needed intra-operatively during prolonged procedures [71]. 

**Recommendation 5.** In the pediatric patient undergoing interventional cardiac catheterization with the placement of prosthetic material, peri-operative prophylaxis with cefazolin is recommended with a single dose of 30 mg/Kg (maximum dose 2 g) IV to be administered in the 30 min before surgery and repeatable if the surgery lasts more than 4 h. 

#### 3.1.4. SCENARIO #4: Implantable Cardiac Defibrillator or Epicardial Pacemaker Placement

The use of implantable electronic cardiac devices in the pediatric population has increased in recent years. Infections related to these devices remain an important cause of mortality and morbidity, with a higher risk of infectious complications than in the adult population [76,77]. A large meta-analysis on adult patients showed the effectiveness of antibiotic prophylaxis during pacemaker placement in preventing infectious complications, thus encouraging its routine use [69]. Subsequently, the meta-analysis by Darouiche’s group also suggested the efficacy of antibiotic prophylaxis administered within 1 h prior to the implantation of an electronic heart device in reducing SSIs [77]. The study by Bertaglia and colleagues supported the use of a single intravenous dose of 2 g cefazolin for the prevention of infectious complications related to pacemaker implantation or replacement [78]. In 2010, the American Heart Association (AHA) published guidelines, also valid for the pediatric population, which recommended the use of a single dose of an antimicrobial with antistaphylococcal activity before device placement, while not recommending the prolongation of prophylaxis in the post-operative period for a lack of data supporting its effectiveness. If the antibiotic of choice is cefazolin, this should be administered intravenously within 1 h before incision, whereas in the case of vancomycin, administration should be within 2 h before incision [79]. 

Clinical practice guidelines for antimicrobial prophylaxis from Bratzler’s group recommend in the adult population the use of antibiotic prophylaxis with a single dose of cefazolin or cefuroxime for pacemaker implantation or replacement, defibrillator implantation, or cardiac resynchronization device [5]. 

**Recommendation 6.** In the pediatric patient undergoing surgical epicardial implantable cardiac defibrillator or pacemaker placement surgery via thoracotomy or sternotomy or subxiphoid or subcostal incision, peri-operative prophylaxis with cefazolin is recommended with a single dose of 30 mg/Kg (maximum dose 2 g) IV to be administered in the 30 min before surgery and repeatable if the surgery lasts longer than 4 h. No post-operative dose is recommended. 

#### 3.1.5. SCENARIO #5: Patients Undergoing ExtraCorporeal Membrane Oxygenation (ECMO)

ECMO is a form of cardiopulmonary bypass in which venous blood is drained outside the patient and circulated in contact with a membrane for gas exchange; the oxygenated blood then re-enters the subject’s body through a vein (called veno-venous or VV ECMO) or an artery (called veno-arterial or VA ECMO). The goal of this technique is to support both the lung and the heart function such that it can provide time for the underlying disease process to resolve [80]. The ECMO technique seems to provide an advantage in terms of management and survival in patients with severe forms of congenital diaphragmatic hernia, whereas in less severe cases the potential complications of the method continue to outweigh the potential benefits [81,82,83,84]. 

ECMO-related infections show wide variability in the literature, ranging from 6% to 30%, and are associated with high mortality [85,86,87]. The predominantly implicated microorganisms are Gram-positive cocci and *Candida* spp., with eventual biofilm formation [88,89]. Kim’s group found a strong correlation between blood infections and the colonization of ECMO catheters [90]. Specifically, Tse-Chang’s group reported that the risk of bloodstream infection correlated with the duration of ECMO. Although the point of origin of infection (in the intravascular catheter, circuit, or another location) was unclear, the authors observed the resolution of systemic infection in three quarters of the children without catheter removal or circuit replacement [91]. Recently, Yeo’s group showed a reduction in the incidence of blood infections and sepsis-related mortality in subjects undergoing ECMO preceded by circuit disinfection with 2% chlorhexidine gluconate +70% isopropyl alcohol [92]. The Butler Group did not identify specific risk factors for the development of bloodstream infections during ECMO and reported no evidence in favor of antibiotic prophylaxis in these subjects [93]. 

The ELSO Infectious Disease Task Force does not recommend the routine use of antibiotic prophylaxis in subjects undergoing ECMO but does recommend prophylaxis in subjects undergoing cardiac surgery, because of an increased risk in this category of infections, particularly mediastinitis. In these subjects, the choice to use antibiotic prophylaxis is based on multiple factors, including the duration of chest opening, the circumstances of surgery (elective or emergency), the immune and nutritional status of the subject, the probability of the contamination of the open wound, and the presence of a pre-existing infection (e.g., MRSA colonization). Any antibiotic prophylaxis should follow the standard principles of SAP, with a single dose of antibiotic and possible repetitions that should, however, not extend beyond 24 h, due to documented lack of benefit [94]. The same Task Force recommends a “cautious, but aggressive” attitude toward the use of an antifungal as prophylaxis, especially in high-risk patients (prolonged chest opening, broad-spectrum therapy, severely immunocompromised individuals) [94]. 

The work of Franzier and colleagues, based on a multicenter electronic questionnaire, showed that 74% of subjects undergoing ECMO had received antibiotic prophylaxis during the procedure (and, in particular, 68% of these only at the time of cannulation, while 24% for the entire duration of the procedure). Despite a reported variety of antibiotics, the group found a prevalent use of cefazolin as monotherapy. Moreover, 18.4% of the patients had also undergone antifungal prophylaxis [95]. 

Previously, Kao’s group also reported a similar rate of antibiotic prophylaxis use in subjects undergoing ECMO; however, they recorded greater variability in molecule use [96]. The work of Farrel and colleagues confirmed the extensive use of antibiotic prophylaxis in children undergoing ECMO, despite the absence of evidence [97]. An interesting study by Adembri and colleagues, albeit with limitations related to the method and small sample size, documented a higher plasma level of cefazolin when administered continuously (instead of in boluses), without an increase in the total dose of drug administered in patients undergoing cardiac surgery using cardiopulmonary bypass [98]. 

Lanckohr’s group analyzed the pharmacokinetics of cefazolin during its use in cardiopulmonary bypass, noting that this was influenced by renal function (creatinine), albumin, and total protein levels. In particular, the authors pointed out that the institution of cardiopulmonary bypass initially increases the volume of distribution, thereby prolonging the half-life of cefazolin. Subsequently, at the end of extracorporeal circulation, there is a reversal of this phenomenon. Also during the course of the procedure, the authors observed a reduction in albumin and total protein levels, resulting in a reduction in the volume of the distribution of cefazolin [99]. 

**Recommendation 7.** In pediatric and neonatal patients undergoing either veno-venous or veno-arterial ECMO, it is recommended to administer the peri-operative prophylaxis with cefazolin with a single dose of 30 mg/Kg (maximum dose 2 g) IV in the 30 min before surgery, repeatable in the case of surgery lasting more than 4 h, associated with any other prophylaxis in place for the specific intervention. It is not recommended to continue prophylaxis for more than 24 h after the end of the procedure. 

#### 3.1.6. SCENARIO #6: Patients Undergoing Other Cardiac Surgery

The treatment of cardiac tumors and heart transplantation are rare procedures, especially in the pediatric population. No specific indications are reported in the literature; this is also because of the complex nature of the procedure itself, which necessarily requires individual evaluation. Some authors focus on the prophylaxis of antiviral and fungal infections, and to a greater extent as secondary prophylaxis, i.e., after the transplantation, considering the immunosuppression of the subject [100,101]. The experts on the panel decided not to provide specific indications in this regard, but to recommend a multi-specialist consultation in this case. 

**Recommendation 8**. In the case of other cardiac surgeries such as the treatment of cardiac tumors and heart transplantation, a multi-specialist consultation is recommended for the establishment of individualized peri-operative antimicrobial prophylaxis.

### 3.2. Non-Cardiac Thoracic Surgery

#### 3.2.1. SCENARIO #7: Non-Cardiac Thoracic Surgery with Thoracotomy 

Table 2 shows the main procedures of non-cardiac thoracic surgery with thoracotomy.

Non-cardiac thoracic surgeries with or without the resection of part of the lung in the absence of a pre-existing infection, excluding esophageal surgery, are classified as clean/contaminated procedures [102]. Because of manipulations of and interventions in the bronchi or trachea during the procedure, pathogens of the oropharyngeal flora, in addition to skin flora, may also colonize the tracheobronchial tree and be responsible for post-operative infections [103]. 

There are currently no guidelines for the use of peri-operative prophylaxis in the pediatric population. Studies in the adult population, many of which are not recent, have demonstrated the efficacy of a single dose of antibiotic prophylaxis in pulmonary surgery [104]. Studies agree on the indication of pre-operative broad-spectrum prophylaxis with cephalosporins, which is considered the standard for prophylaxis in pulmonary surgery due to its efficacy against frequently involved pathogens, low cost, and low allergenic potential [105,106]. Bratzler’s group’s guidelines for the adult patient recommend cefazolin or, alternatively, the combination of ampicillin and sulbactam [5].

It should be emphasized that, in thoracic surgery, in addition to SSIs, empyema and post-operative nosocomial pneumonia also represent a real infectious risk, which antibiotic prophylaxis with cefazolin alone does not seem effective in preventing [5,105,106]. Reported cases of SSIs, empyema, and post-operative pneumonia following elective thoracic surgery range from 7% to 14% [107]. Some studies have specifically addressed the relationship between peri-operative prophylaxis for SSI prevention and the prevention of empyema and post-operative pneumonia. A randomized controlled trial, conducted by Bernard’s group, claimed that prolonged antibiotic use, up to 48 h after surgery, was able to decrease the incidence of empyema in the post-operative period, from 15.6% with two doses to 6% with 48 h of treatment. However, the result could be affected by complications that arose in the first group due to the surgical technique and not by the use of prophylaxis [108]. Radu and colleagues stated that peri-operative prophylaxis aimed at preventing SSIs is inefficient toward postoperative pneumonia in cases of large lung resections [103]. Subsequently, the work of Schussler and colleagues demonstrated that SAP targeting the colonizing bacteria of the bronchial tree could be successful in preventing post-operative pneumonia, where the use of second-generation cephalosporins alone is insufficient [109]. 

**Recommendation 9.** In the pediatric and neonatal patient undergoing thoracic, non-cardiac surgery by thoracotomy, peri-operative prophylaxis with cefazolin is recommended with a single dose of 30 mg/Kg (maximum dose 2 g) IV to be administered within 30 min before surgery and repeatable if the surgery lasts longer than 4 h. 

#### 3.2.2. SCENARIO #8: Non-cardiac Thoracic Surgery Using Video-Assisted Thoracoscopy

Video-assisted thoracoscopic (VAT) surgery accounts for approximately one-third of all thoracic surgical procedures (Table 3). 

VAT is a technique that involves small incisions, resulting in a lower rate of SSI when compared to that associated with open thoracic procedures, estimated at 1.7% after minimally invasive procedures, according to the extensive review by Solaini’s group [110,111]. This work also identifies chronic obstructive pulmonary disease as a major risk factor for infection in adults, thus recommending the administration of peri-operative antibiotic prophylaxis for this category [111]. There are no universally accepted guidelines or randomized controlled clinical trials in the pediatric age regarding peri-operative antibiotic prophylaxis during thoracic surgery using VAT. The joint guidelines of the American Society of Health-System Pharmacists, the Infectious Diseases Society of America, the Surgical Infection Society, the Society for Healthcare Epidemiology of America, and the European Society of Thoracic Surgeons propose a single recommendation for the adult population that applies to both thoracotomy and VAT surgery [5]. They recommended prophylaxis with cefazolin or ampicillin and sulbactam, with recommendation grade A in thoracotomy and recommendation grade C in VAT surgery [5]. 

**Recommendation 9.** In the pediatric and newborn patient undergoing non-cardiac thoracic surgery by video-assisted thoracoscopy (VAT), peri-operative prophylaxis with cefazolin is recommended with a single dose of 30 mg/Kg (maximum dose 2 g) IV to be administered within 30 min before surgery and repeatable if the surgery lasts longer than 4 h.

#### 3.2.3. SCENARIO #9: Elective Chest Drain Placement in the Pediatric Patient

Chest drain placement is a procedure that is considered clean/contaminated with a relatively low risk of infection. Major indications for thoracostomy placement include: pneumothorax, penetrating or blunt chest trauma, hemothorax, chylothorax, empyema, and the post-operative period of thoracic or cardiac surgery [112]. Currently, there are no clinical studies available concerning the pediatric or neonatal population regarding preoperative antibiotic prophylaxis in the case of elective chest drain placement. Moreover, as in adult patients, the available data mainly concern the trauma setting, which is associated, however, with a higher infectious risk secondary to wound contamination. 

A randomized clinical trial conducted in 245 adults demonstrated that extending post-operative prophylaxis (for 48 h or until drain removal) does not appear to offer any additional benefit in terms of reducing infectious complications compared with standard antibiotic prophylaxis. Even more, it could potentially be associated with more adverse effects, such as the selection of more resistant micro-organisms, allergic reactions, drug toxicity, *Clostridium difficile* colitis, and higher costs [107]. An observational study conducted on 119 patients with spontaneous pneumothorax treated with elective thoracostomy did not highlight the need for peri-procedural antibiotic prophylaxis, since the elective procedure is performed in a sterile environment and the duration of drainage is less than a week [113]. Regarding patients admitted to the intensive care unit, there are no studies regarding peri-procedural antibiotic prophylaxis and recommendations are often based on data from trauma patients [114]. The British Thoracic Society considers elective chest drain placement in spontaneous pneumothorax to be a procedure with a low risk of developing an infectious complication and recommends the administration of antibiotic prophylaxis only if there is concomitant trauma [115]. Similarly, the guidelines of the French Society of Anesthesia and Resuscitation do not recommend the administration of any prophylaxis not only in the case of the placement of chest drainage, but also in the case of mediastinoscopy, videothoracoscopy, and thoracoscopy [116]. 

**Recommendation 10.** In the pediatric patient (not newborn) undergoing elective chest drain placement, in the absence of a pre-existing infection, peri-operative antibiotic prophylaxis is not recommended. 

#### 3.2.4. SCENARIO #10: Elective Chest Drain Placement in the Newborn

Neonates, especially those with a low birth weight or premature infants, commonly require the placement of an intercostal drain in cases of intrathoracic collections, the presence of pneumothorax, or pleural effusion. Low-birth-weight or premature infants have compromised local and systemic defense mechanisms. In these patients, the intercostal tube represents a potential risk factor for nosocomial infections because of the skin barrier breakdown [117]. Infection secondary to the use of intercostal catheters could cause a significant increase in mortality and morbidity, with a prolonged hospital stay, increased severity of respiratory disease, and unfavorable neurological outcomes. With this in mind, newborns might benefit from peri-procedural antibiotic prophylaxis, considering the potential increase in side effects related to antibiotic misuse. Patel’s group, in a multicenter study evaluating the inappropriate use of antibiotics in the neonatal population, reported the routine use of antibiotic prophylaxis for intercostal catheter insertion, although without observing evidence of benefit [118]. 

A 2012 systematic review, in view of the lack of randomized controlled trials regarding intercostal drain placement in the neonatal population, concluded that there was a lack of evidence to support or refute the routine use of antibiotic prophylaxis in the neonatal population [117]. Randomized trials specific to the neonatal population are needed, which in this case should take into account that infants requiring intercostal catheter insertion may already be receiving antibiotics for other indications. 

**Recommendation 11.** In the infant undergoing elective chest drain placement surgery in the absence of pre-existing infection, peri-operative antibiotic prophylaxis is not recommended. 

#### 3.2.5. SCENARIO #11: Thoracic Drain Placement in the Trauma Setting

The placement of a pleural drain is often the first step in the management of blunt or penetrating chest trauma. The placement of a thoracostomy tube is considered necessary, especially in cases of pleural rupture with pneumothorax, intra-pleural bleeding causing hemothorax, or in cases of hemo-pneumothorax [119,120]. Because these procedures are frequently performed on an emergency basis, peri-procedural hygiene conditions are often not optimal. As a result, contamination during drain insertion represents a major cause of the development of infectious complications, including wound infections, pneumonia, and empyema [121]. The organisms responsible for infection vary depending on the mechanism of contamination. The most frequently involved are pathogens present on the penetrating foreign body or on the skin, such as *S. aureus* or other Gram-positive bacteria, while contamination by Gram-negative or mixed bacteria are secondary to pulmonary processes or other routes of spread [122,123]. Infectious complications, and in particular empyema, occur more frequently after penetrating (as opposed to blunt) chest trauma, because penetrating injuries allow the direct entry of micro-organisms into the pleural cavity [112]. 

Currently, no consistent data are available in the pediatric population regarding the administration of antibiotic prophylaxis in case of chest drain placement following trauma. Moreover, even in adult patients this topic is rather debated. Some studies conducted in the adult population, although dated, supported the use of antibiotics before and after the procedure without defining how long antibiotics should be administered to prevent complications such as empyema and pneumonia [124,125,126,127,128,129,130]. On the other hand, two large prospective randomized trials compared the use of antibiotics versus placebo in patients with thoracostomies, without finding significant differences in the incidence of infectious complications between the two groups and, therefore, not supporting the administration of antibiotic prophylaxis [131,132]. Thus, it emerges that the evidence supporting the use of antibiotic prophylaxis for chest drain placement is quite mixed [133]. 

However, a recent meta-analysis conducted on 1877 patients drew attention to the importance of administering antibiotic prophylaxis in patients with penetrating chest trauma in order to decrease the incidence of infectious complications [134]. Although Ayoub and colleagues reported a strong indication for the administration of antibiotic prophylaxis in these patients, they emphasized the need for future studies that can comprehensively define not only the type of antibiotic, but also the duration of prophylaxis [135]. The British Thoracic Society (BTS) guidelines, in agreement with the Eastern Association for the Surgery of Trauma (EATS) guidelines, strongly recommend the prophylactic administration of antibiotics, despite the low overall incidence of infectious complications after chest drain insertion. In particular, EATS recommends the administration of first-generation cephalosporins (cefazolin), active against *S. aureus*, the main culprit of post-traumatic empyema, with a duration of no more than 24 h [122,136]. 

**Recommendation 12.** In the pediatric patient undergoing surgery for the placement of chest drainage in a trauma setting, peri-operative prophylaxis with cefazolin at a dose of 30 mg/Kg (maximum dose 2 g) IV is recommended to be administered within 30 min before surgery and repeatable in case of surgery lasting more than 4 h.

## 4. Discussion

In general, the recommendations in the literature regarding antibiotic prophylaxis in pediatric cardiothoracic interventions are derived from studies conducted in the adult population. While recognizing a possible higher incidence of SSI in pediatric and neonatal age, usually, in agreement with indications in adults, a prolonged duration of surgical prophylaxis is not used [27,48,137,138,139]. Furthermore, the duration should not depend on the placement of chest drains or vascular or intracardiac catheters [30,138]. However, due to the absence of a high level of evidence for neonatal- and pediatric-age patients, the choice by each clinician can be individualized according to the situation.

In neonates undergoing cardiac surgery, there are no specific recommendations, but those in force in the adult population are generally applied, although the neonatal population seems to be at higher risk of developing SSIs after cardiac surgery [13,140,141]. Murray’s group recommended also following the indications for the adult population with regard to the duration of prophylaxis, stressing the need for specific multicenter studies on this population [138]. 

Patients undergoing cardiac surgery with sternotomy or thoracotomy are often considered at high risk of developing SSIs. Our panel concluded by suggesting the use of cefazolin, as was also proposed by Jaworski [30], emphasizing that there was no justification for extending prophylaxis beyond 24 h after surgery. 

With regard to cardiac catheterization procedures, the decision of whether or not to administer surgical antibiotic prophylaxis depends on the placement of prosthetic material. In fact, cardiac catheterization is considered a “clean” procedure with a low risk of infectious complications. It has been widely demonstrated that the placement of foreign material predisposes to a greater onset of infections, affecting not only the surgical site but, above all, the implanted device. Therefore, the panel of experts concluded by recommending the administration of peri-procedural antibiotic prophylaxis in the case of cardiac catheterization only when the placement of prosthetic material is planned. The molecules of choice remain the first-generation cephalosporins and especially cefazolin, both for their efficacy against the pathogens usually responsible for infectious complications after cardiac catheterization and for reasons of cost, safety, and duration of action [5,12,30]. 

In pacemaker placement, the indications provided by the panel of experts for prophylaxis followed those proposed by the AHA of a single dose of antibiotic before device placement, with no need to prolong administration in the post-operative time [79]. The molecule chosen was cefazolin, plausibly because of its efficacy against the most frequent germs responsible for SSIs. However, a recent survey highlighted a poor adherence to the recommendations in the pediatric setting. In particular, 88% of the 78 physicians interviewed declared that they did not follow the recommendations of the AHA, although 69% admitted that they knew these guidelines. The reasons for non-adherence varied, from personal experience to a fear of increased infectious risk for children compared with adults [76]. From the same work, 81% of participants said that specific recommendations for the pediatric patient would encourage a change in their clinical practice [76]. 

Regarding prophylaxis during interventions which make use of an ECMO procedure, the panel agreed on a routine prophylaxis with cefazolin, emphasizing the need to evaluate individually the different types of intervention. Moreover, some works have emphasized the influence of cardiopulmonary bypass on the pharmacokinetics of antibiotics used peri-operatively in cardiac surgery [20]. These data support the need for the careful monitoring of the plasma dosages of the drug used for intra-operative prophylaxis, also making use of specific alert systems that provide reminders of the need to administer a new dose to improve compliance towards re-dosing [142,143]. 

In patients undergoing thoracotomy and VATS procedures, the expert panel concluded with the advice to perform antibiotic prophylaxis with cefazolin, despite the low incidence of SSIs in thoracoscopy regimens. This prophylaxis does not seem to be sufficient in reducing the incidence of pneumonia and empyema, which should be considered as SSIs in thoracic surgery procedures [5,110]. 

For the elective placement of chest drainage, in agreement with the current suggestions for adults, the expert panel decided to not recommend antibiotic prophylaxis in the case of chest drainage [122,123]. However, further studies are needed regarding the neonatal population in particular, which has, on the one hand, an increased risk of developing infectious complications with consequent increased morbidity and mortality and, on the other hand, may already be undergoing antibiotic therapy for other problems [124]. 

Regarding the placement of chest drainage in cases of penetrating or blunt trauma, the role of prophylactic antibiotics is somewhat controversial. While the value of antibiotic prophylaxis in surgical procedures is supported by many studies, its use in trauma and wound patients is less clear. In fact, there is often no way to administer an antibiotic before bacterial contamination occurs, so the drugs used in this context are traditionally administered at therapeutic dosages for early ‘presumptive’ therapy, and not, therefore, for properly prophylactic purposes. The aim of this therapy is the same as prophylaxis, i.e., to reduce the incidence of infectious complications after a therapeutic intervention [129]. In this context, attention must also be paid to the use of a technique for tube placement that is as aseptic as possible [143]. In addition, while there appears to be a greater reduction in the risk of infection with lower morbidity and mortality, the effect of antibiotic prophylaxis for thoracostomy in penetrating trauma is still uncertain [130]. On the basis of the available literature, the panel decided to recommend the use of antibiotic prophylaxis with agents active against *S. aureus* when placing chest drains in patients with trauma to reduce the risk of wound infection, pneumonia, or empyema. 

Patients undergoing cardiac and thoracic surgery may present specific high-risk conditions, including colonization by methicillin-resistant *Staphylococcus aureus* and multidrug resistant bacteria, allergy to first-line antibiotics, underlying immunodeficiency, already receiving antibiotic therapy or prophylaxis for other reasons, or having an infection in sites other than the surgical site. For these conditions, we developed a specific consensus document with the aim to respond to issues that are still little-addressed with specific scenarios developed to guide the healthcare professional in practice [144].

Through the RAND method, the participants in our study discussed statements derived from the guidelines, and an agreement was reached as to the recommendations for surgical antimicrobial prophylaxis in cardiac and non-cardiac thoracic surgery. It should be noted that the participants in the project came from different clinical contexts, i.e., they were pediatricians, neonatologists, infectious disease specialists, pediatric cardiac surgeons, pediatric thoracic surgeons, anesthetists, pharmacologists, and microbiologists. For this reason, the results achieved demonstrate the usefulness of the RAND method for the selection of good practices and constitute the basis of an evidence-based approach. The findings obtained can establish the basis for educational interventions that aim to optimize the use of antibiotics in pediatric patients undergoing cardiac surgery and thoracic surgery. The limitations of the study included that this was an opinion-based survey to produce recommendations, and the agreement was reached in a collegial meeting. On the other hand, the lack of pediatric studies on the topic did not permit the use of the GRADE methodology, and the complexity of the topic required an online face-to-face meeting with all the participants. However, the RAND method did not permit the definition of a hierarchy of antibiotic administration, and not using the GRADE method may have affected the quality of these recommendations.

## 5. Conclusions

Patients undergoing cardiac surgery and thoracic surgery are often considered special patients and more at risk of developing SSIs, so they often undergo peri-operative antibiotic prophylaxis on different schedules, which is not always supported by scientific evidence. This consensus provides clear and shared indications, based on the most up-to-date literature. 

This work has been made possible by the multidisciplinary contribution of experts belonging to the most important Italian scientific societies and represents, in our opinion, the most complete and up-to-date collection of recommendations on the behavior to be applied in the peri-operative setting in this type of intervention, in order to guide physicians in the management of the patient, standardizing approaches and avoiding the abuse and misuse of antibiotics. Table 4 and Table 5 summarize the recommendations. The dosage and timing are those recommended for normal renal function; dose adjustments (i.e., reduction in dose and/or frequency of administration) should be considered in the case of renal impairment. Recommendations for patients with allergies to penicillin are summarized in another article of our study group [144]. These recommendations would serve the further development of evidence-informed enhanced recovery protocols relative to antimicrobial prophylaxis and can ultimately lead to improved peri-operative care across pediatric surgical specialties [145,146].

## Figures and Tables

**Table 1:** Major congenital heart diseases.

Congenital Heart Diseases
Patent ductus arteriosus
Ostium primum type atrial septal defect
Ostium secundum type atrial septal defect
Partial anomalous pulmonary venous connection
Total anomalous pulmonary venous connection
Ventricular septal defect
Partial/Complete atrial septal defect
Atrioventricular septal defect
Tetralogy of Fallot
Transposition of great arteries
Double outlet right ventricle
Pulmonary stenosis
Aortic valve stenosis
Coarctation of the aorta
Univentricular heart
Hypoplasia of pulmonary arteries
Hypoplasia of the aortic arch

**Table 2:** Main procedures of non-cardiac thoracic surgery with thoracotomy.

Non-Cardiac Thoracic Surgery
Primary lung tumors (rare) and metastatic tumors (more common)
Congenital lung malformations
Congenital vascular malformation of the chest
Congenital chest deformities

**Table 3:** Indications for non-cardiac thoracic surgery using video-assisted thoracoscopy.

Non-Cardiac Thoracic Surgery Using Video-Assisted Thoracoscopy
Lobectomy and segmentectomy (congenital lung lesions)
Thoracoscopic biopsies
Congenital diaphragmatic hernia
Congenital esophageal atresia
Congenital esophageal duplications
Congenital vascular malformation
Bronchogenic cyst
Congenital chest deformities (pectus excavatum)
Pulmonary empyema
Pulmonary metastasectomy for oligometastatic disease

**Table 4:** Summary of recommendations for cardiac surgery.

Cardiac Surgery	Prophylaxis	Molecule	Dosage and Timing
Correction of congenital heart disease and/or valve surgery, with sternotomy or thoracotomy	YES	Cefazolin	- Single dose of 30 mg/Kg (maximum dose 2 g) IV, within 30 min before surgery (Recommendation 1) - Repeat at a dose of 30 mg/Kg (maximum dose 2 g) IV in case of intervention lasting more than 4 h (Recommendation 2) - Continue prophylaxis with cefazolin at a dose of 30 mg/Kg (maximum dose 2 g) IV every 8 for 24 h after sternum closure (Recommendation 3)
Diagnostic or interventional cardiac catheterization without prosthetic material placement	NO	-	-
Interventional cardiac catheterization with prosthetic material placement	Yes	Cefazolin	- Single dose of 30 mg/Kg (maximum dose 2 g) IV, in the 30 min before surgery and repeatable in case of surgery lasting more than 4 h
Placement of implantable cardiac defibrillator or epicardial pacemaker (PM), with thoracotomy or sternotomy or subxiphoid or subcostal incision.	Yes	Cefazolin	- Single dose of 30 mg/Kg (maximum dose 2 g) IV, in the 30 min before surgery and repeatable in case of surgery lasting more than 4 h
Patient undergoing extracorporeal circulation (ECMO), both venous and veno-arterial	Yes	Cefazolin	- Single dose of 30 mg/Kg (maximum dose 2 g) IV, in the 30 min before surgery and repeatable in case of surgery lasting more than 4 h, to be continued for no more than 24 h after the end of the procedure - Association with any prophylaxis in place for the specific intervention
Other interventions (treatment of cardiac tumors and heart transplantation)	Yes		- A multi-specialist consultation is recommended for the definition of a personalized peri-operative antimicrobial prophylaxis

**Table 5:** Summary of recommendations for thoracic surgery.

Non-Cardiac Thoracic Surgery	Prophylaxis	Molecule	Dosage and Method of Administration
Non-cardiac thoracic surgery with thoracotomy	Yes	Cefazolin	Single dose of 30 mg/Kg (maximum dose 2 g) IV, within 30 min before surgery, repeatable if surgery lasts more than 4 h
Non-cardiac thoracic surgery using video-assisted thoracoscopy (VATS)	Yes	Cefazolin	Single dose of 30 mg/Kg (maximum dose 2 g) IV, within 30 min before surgery, repeatable if surgery lasts more than 4 h
Elective placement of chest drainage in pediatric patients	NO	-	-
Elective chest drain placement in neonatal age patients	NO	-	-
Placement of chest drainage in the traumatology field	Yes	Cefazolin	Single dose of 30 mg/Kg (maximum dose 2 g) IV, within 30 min before surgery, repeatable if surgery lasts more than 4 h

## Data Availability

All the data are included in the manuscript.
